# Wounds in War

**Published:** 1899-03

**Authors:** 


**Wounds in War.** By Surgeon-Colonel W. F. STEVENSON, M.B. Pp. xv., 419. London: Longmans, Green, and Co. 1897.

It is with great satisfaction and interest that we have read this book of Surgeon-Colonel Stevenson's, and we welcome it cordially, for no one should be more qualified to speak on the subject than the Professor of Military Surgery at Netley. Practically, as Colonel Stevenson points out, the subject is limited to gunshot wounds, those inflicted by sword or bayonet being, at least in warfare between civilised nations, a negligable quantity. Not that our author has by any means omitted to mention other injuries, but he has given them only a small share of his consideration proportionate to their importance. In his clearly printed pages, abundantly illustrated and well furnished with diagrams and tables, he has embodied a vast store of information useful to the military surgeon, and by no means wanting in interest to his civilian *confrère*. Each paragraph is headed with its subject in heavy black type, a plan which increases the value of the book as a work of reference.

The first chapter deals with the mechanism of projectiles, their motion and velocity, and the effects upon them of the resistance of the air and gravitation. It concludes with a description of the Lee-Metford magazine rifle, which has been described as "the best military small arm in existence." We record this fact with some amount of pride, as the part-inventor of this weapon is a Bristol citizen. The second chapter details the characteristics of injuries produced by different projectiles —by the old round bullet, the modern cylindro-conoidal bullet, and by large projectiles and their fragments.

A short chapter is devoted to the primary symptoms of wounds, namely, pain, local shock, anæsthesia, hemorrhage, and the treatment of these conditions, followed by a consideration of the general treatment of wounds in war, with an interesting contrast between the old and new methods, and the bacteriology of wounds. Under the heading "First Field Dressings," great stress is laid upon the importance of the handling of a wound as little as possible, in order to avoid the introduction of septic material.

We are now introduced to the more special subject of the book, namely, gunshot wounds and their treatment. Our author, after again emphasising his remarks on the non-interference with wounds on the field, describes the various means of detecting the extent of injury, and the presence of a bullet or other foreign substance. The finger of the surgeon, Nélaton's probe, or other more clumsy contrivance for diagnosis, will, for the future, be superseded by Röntgen's marvellous discovery, and one incalculable source of danger to the wounded soldier avoided. The risk of secondary hemorrhage, an accident far less common than it used to be, and caused by disintegration of the wall of the vessel by septic arteritis, is minimised by the introduction of the antiseptic treatment of wounds.

From the general consideration we pass to a most interesting and carefully compiled description of the injuries of individual structures of the different joints, the long bones, head, thorax, and abdomen, the indications for conservative treatment, for amputation, and cautions in dealing with penetrating wounds of lung and brain. All these are of great practical value.

The ninth chapter deals with wounds of the abdomen. Penetrating wounds of the abdomen are by far the most fatal of all injuries in war, the mortality running as high as 90 per cent.

It would, we think, be advantageous to found upon the details given in these valuable chapters a small handbook of aphorisms which could be regarded as authoritative, and would, if well put together, be a safe guide and help to the military surgeon in those formidable emergencies with which he may be brought into contact.

The thirteenth chapter is devoted firstly to statistics, and secondly to a description of the duties of bearers in the field, the first dressings, transport of the wounded, and the management of the field hospital, whilst the concluding chapter gives us a *résumé* of the work of the Geneva Convention, and expresses a hope that its suggestions will in time meet with a more general appreciation, and that the Red Cross will be universally recognised as a military badge of neutrality.

